# A Quality Control Mechanism Linking Meiotic Success to Release of Ascospores

**DOI:** 10.1371/journal.pone.0082758

**Published:** 2013-12-02

**Authors:** Haiyan Guo, Megan C. King

**Affiliations:** Department of Cell Biology, Yale School of Medicine, New Haven, Connecticut, United States of America; Texas A&M University, United States of America

## Abstract

Eukaryotic organisms employ a variety of mechanisms during meiosis to assess and ensure the quality of their gametes. Defects or delays in successful meiotic recombination activate conserved mechanisms to delay the meiotic divisions, but many multicellular eukaryotes also induce cell death programs to eliminate gametes deemed to have failed during meiosis. It is generally thought that yeasts lack such mechanisms. Here, we show that in the fission yeast *Schizosaccharomyces pombe*, defects in meiotic recombination lead to the activation of a checkpoint that is linked to ascus wall endolysis – the process by which spores are released in response to nutritional cues for subsequent germination. Defects in meiotic recombination are sensed as unrepaired DNA damage through the canonical ATM and ATR DNA damage response kinases, and this information is communicated to the machinery that stimulates ascus wall breakdown. Viability of spores that undergo endolysis spontaneously is significantly higher than that seen upon chemical endolysis, demonstrating that this checkpoint contributes to a selective mechanism for the germination of high quality progeny. These results provide the first evidence for the existence of a checkpoint linking germination to meiosis and suggest that analysis solely based on artificial, enzymatic endolysis bypasses an important quality control mechanism in this organism and potentially other ascomycota, which are models widely used to study meiosis.

## Introduction

Meiosis lies at the heart of sexual reproduction, encompassing programmed recombination between homologous chromosomes followed by two rounds of specialized cell division that gives rise to haploid gametes or spores. A prerequisite to successful meiotic recombination is the pairing of homologous chromosomes. In the fission yeast, *Schizosaccharomyces pombe*, homologue pairing during meiotic prophase involves formation of the telomeric bouquet, in which the telomeres become associated with the spindle pole body [1], and a pronounced period of nuclear oscillations termed “horsetail motion” [2], [3]. Recombination between homologous chromosomes requires programmed DNA double strand breaks (DSBs) induced by the enzyme Rec12, the *S. pombe* homologue of Spo11, and DNA repair dependent on a specialized homologous recombination pathway that promotes meiotic crossovers [4].

Observations in a broad array of eukaryotic models suggests that meiosis is an error-prone process, requiring numerous checkpoints to ensure successful gametogenesis either through delay of the meiotic divisions or, in higher eukaryotes, the removal of failed meiotic products through apoptotic pathways [5]. A failure to cull gametes leads to infertility and/or genetic disorders in humans [6]. Established meiotic checkpoints link formation of DSBs to successful replication, monitor the repair of Spo11-induced DSBs, and assess the fidelity of chromosome pairing [7]. The model yeast *Saccharomyces cerevisiae* is capable of delaying the meiotic divisions in response to such stimuli, but display checkpoint adaptation that ultimately permits spore formation even in the face of persistent DNA damage [8]. Further, *S. pombe* either lack or have a very weak recombination checkpoint response compared to *S. cerevisiae* [9], [10]. It is unclear whether yeasts possess mechanisms to cull progeny resulting from a failed meiosis analogous to the apoptotic pathways employed by multicellular eukaryotes.

In *S. pombe*, conjugation of cells of the opposite mating type gives rise to the ascus, the cell wall that will encapsulate the four ascospores arising from meiosis resulting in so-called “tetrads” [11]. Prior to germination, the ascus wall, made predominantly of α- and β-glucans [12], must be broken down to release the ascospores. In *S. pombe*, release of ascospores is achieved through an active process of ascus wall endolysis, which requires at least two glucanases, encoded by the *agn2* [13] and *eng2* [14] genes. Little is known about how these gene products are regulated, although their expression has been reported to increase with the “middle genes” during sporulation [15].

Here, we show that certain types of meiotic defects lead to the activation of a checkpoint that inhibits release of ascospores from the ascus wall, potentially preventing spores generated by an aberrant meiosis from competing for resources with high-quality progeny or participating in further rounds of mating. This checkpoint is activated in *S. pombe* by meioses that occur in the absence of Kms1 [16], [17], a member of the KASH (Klarsicht, Anc1, SYNE1 homology) protein family. KASH proteins reside in the outer nuclear membrane and indirectly link the telomeres to microtubules during meiotic prophase as part of the LINC (Linker of Nucleoskeleton and Cytoskeleton) complex in most eukaryotes [18]. LINC complex components are broadly required for meiosis [19], as mice lacking SUN1 or its cognate KASH protein, KASH5, are infertile [20], [21] and Matefin/SUN-1 and the KASH protein ZYG-12 are required for proper homologue pairing in *C. elegans* [22], [23]. In these multicellular eukaryotes, loss of meiotic LINC complex function causes massive apoptosis in the germ line, suggesting that control over ascus wall endolysis might represent an *S. pombe* corollary to the apoptotic response.

## Methods

### Strain Generation

All *S. pombe* strains are listed in Table S1. Knock-out strains were generated by gene replacement, as described [24]. Double-knockout strains were generated by genetic crosses followed by marker and mating-type analysis.

### Meiotic crosses

Meiosis was induced by mixing strains of opposite mating types (h+ and h−) on, or plating h90 strains to, malt extract agar plates, followed by incubation at room temperature for 48 hours.

### Analysis of ascus wall endolysis

Tetrads were resuspended in water and plated to rich media (YE5S) plates. Individual tetrads arising from a heterothallic meiosis with four visible spores were micromanipulated into an eight by eight grid using a micromanipulator (Singer MSM). Plates were then incubated at 30°C. After incubation for 24 and 48 hours, plates were returned to the microscope to observe whether ascus wall endolysis had taken place. Values are presented as averages with their standard deviations from at least three replicates each containing 64 tetrads. Statistics were evaluated by unpaired t-test.

### Spore germination and viability measurements

Chemical breakdown was achieved by incubation of meiotic products in 5% beta-glucuronidase (MP Biochemicals) overnight at room temperature to kill vegetative cells and release spores from the ascus walls. After washing three times in water, spores were plated and individual spores were either micromanipulated into eight by eight grids on YE5S plates (for colony forming ability four days later, values are averages of at least three replicates each analyzing 64 spores plotted with their standard deviations) or plated onto YE5S for observation of germination (at 24 hours). In all cases plated spores were incubated at 30°C for the time indicated. To monitor germination, plates were returned to the dissection microscope after 24 hours and spores were visually assessed for their ability to undergo germination as indicated by morphology change and an increase in size. For germination assays, at least three replicates each containing greater than 100 spores were analyzed and data are presented as averages with their standard deviations. For spontaneous ascus wall breakdown, individual tetrads were micromanipulated into two rows of eight per YE5S plate and allowed to breakdown at 30°C. Plates were examined several times over the next 24 hours and released ascospores were dissected to individual positions revealing the fraction of released ascospores that formed colonies four days later. Values are from three replicates each containing at least 12 tetrads that broke down spontaneously (to give 48 possible viable spores).

## Results

### A subset of meiotic defects inhibit ascus wall endolysis

**Figure 1:**
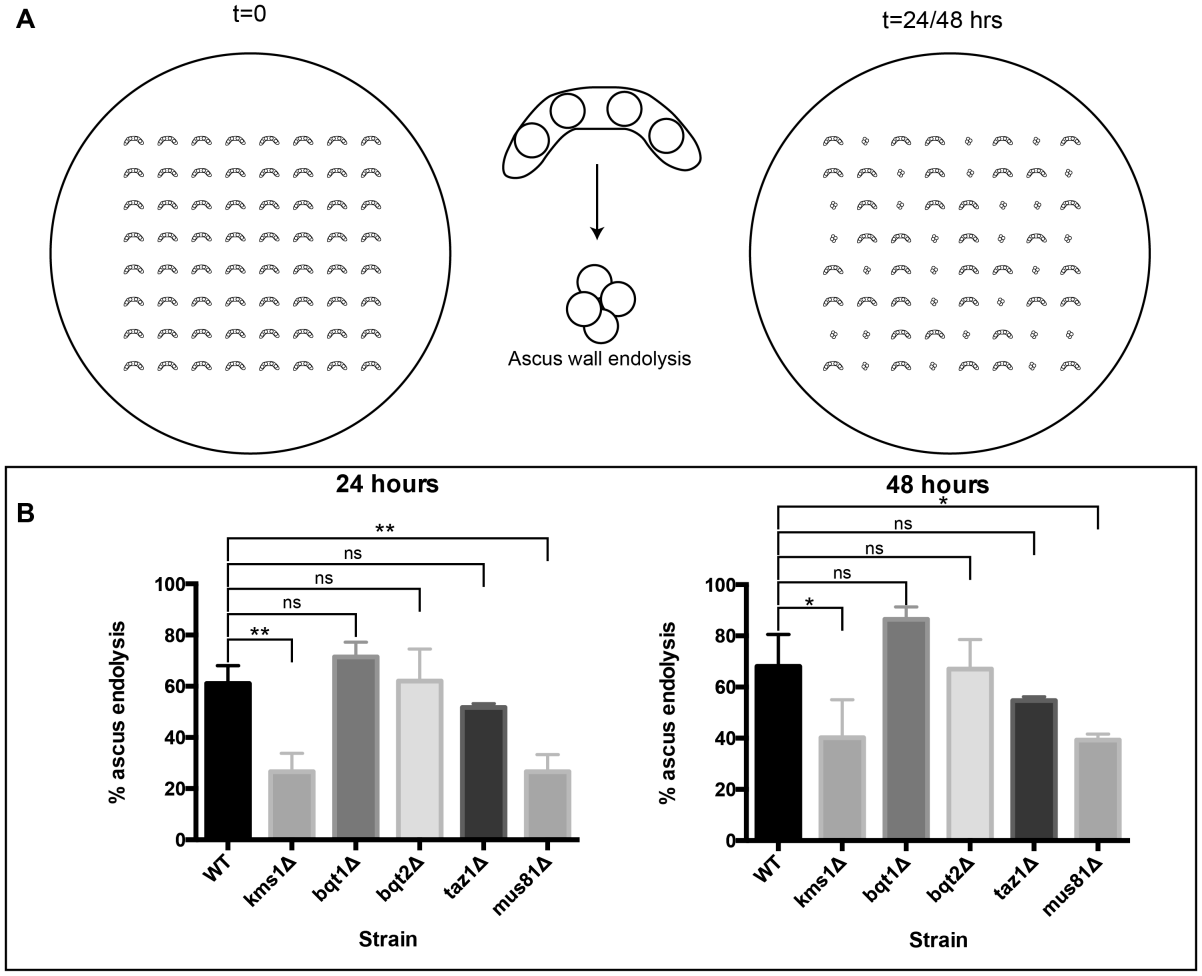
Ascus wall endolysis is inhibited upon perturbation of a subclass of meiotic processes. A. Diagram of the assay for ascus wall endolysis. Individual tetrads are micromanipulated into an 8 by 8 grid on rich media plates. After 24 and 48**B**. Knock-out of Kms1 and Mus81, but not Bqt1 or Bqt2, prevents ascus wall endolysis. Percentage of tetrads from the indicated strain crosses that underwent ascus endolysis was observed at 24 hours (left) and 48 hours (right). Results are derived from at least three experiments and are plotted as the average with its associated standard deviation. p values were determined by unpaired t-test. * p<0.02 ** p<0.004

We made the observation that in some genetic backgrounds where meiotic success is compromised, tetrads underwent ascus wall endolysis at lower rates than wild type (WT) cells. To investigate this more thoroughly, we devised a simple assay in which individual tetrads are micromanipulated onto an eight by eight grid on a rich media plate and allowed to germinate at 30°C. By observing the plates over several days, the number of tetrads that have undergone ascus wall endolysis can be visually counted (Figure 1A).

Using this assay, we found that approximately 60 percent of WT tetrads undergo ascus wall endolysis within 24 hours; this percentage did not appreciably increase at 48 hours (Figure 1B). Ultimately about 85% of WT tetrads gave rise to colonies after 96 hours, suggesting that some tetrads do breakdown after the 48 hour time point. By contrast, tetrads arising from the mating of *kms1Δ* cells had nearly 3-fold less ascus wall endolysis at 24 hours (just over 20%), although this increased to about 40% by 48 hours. Because Kms1 has been implicated in telomere bouquet formation and horsetail motion, we investigated other proteins required for these processes. Interestingly, loss of Bqt1 or Bqt2, which are essential for bouquet formation [25], had no appreciable effect on the rate of ascus wall endolysis. Association of the Bqt proteins with Sad1, which provides the physical link to Kms1, requires the telomere-binding protein Taz1 [25]. *taz1Δ* cells had a reproducible decrease in the rate of ascus wall endolysis, but this did not reach statistical significance (Figure 1B, p = 0.087 at 24 hours).

Since mutations affecting bouquet formation had little effect on the rate of ascus wall endolysis, we wondered if defects in meiotic recombination, which also lead to a delay in the meiotic divisions, might impinge on ascus wall endolysis. Consistent with this idea, the absence of Mus81, which is essential for resolving meiotic crossovers [26], [27], leads to rates of ascus wall endolysis that mimic those seen for *kms1Δ* (Figure 1B). This suggests that pathways that stimulate delays in meiotic division might also inhibit or delay ascus wall endolysis, even though this process takes place days after spores are generated.

### Preventing meiotic DNA double strand breaks suppresses the endolytic checkpoint

**Figure 2:**
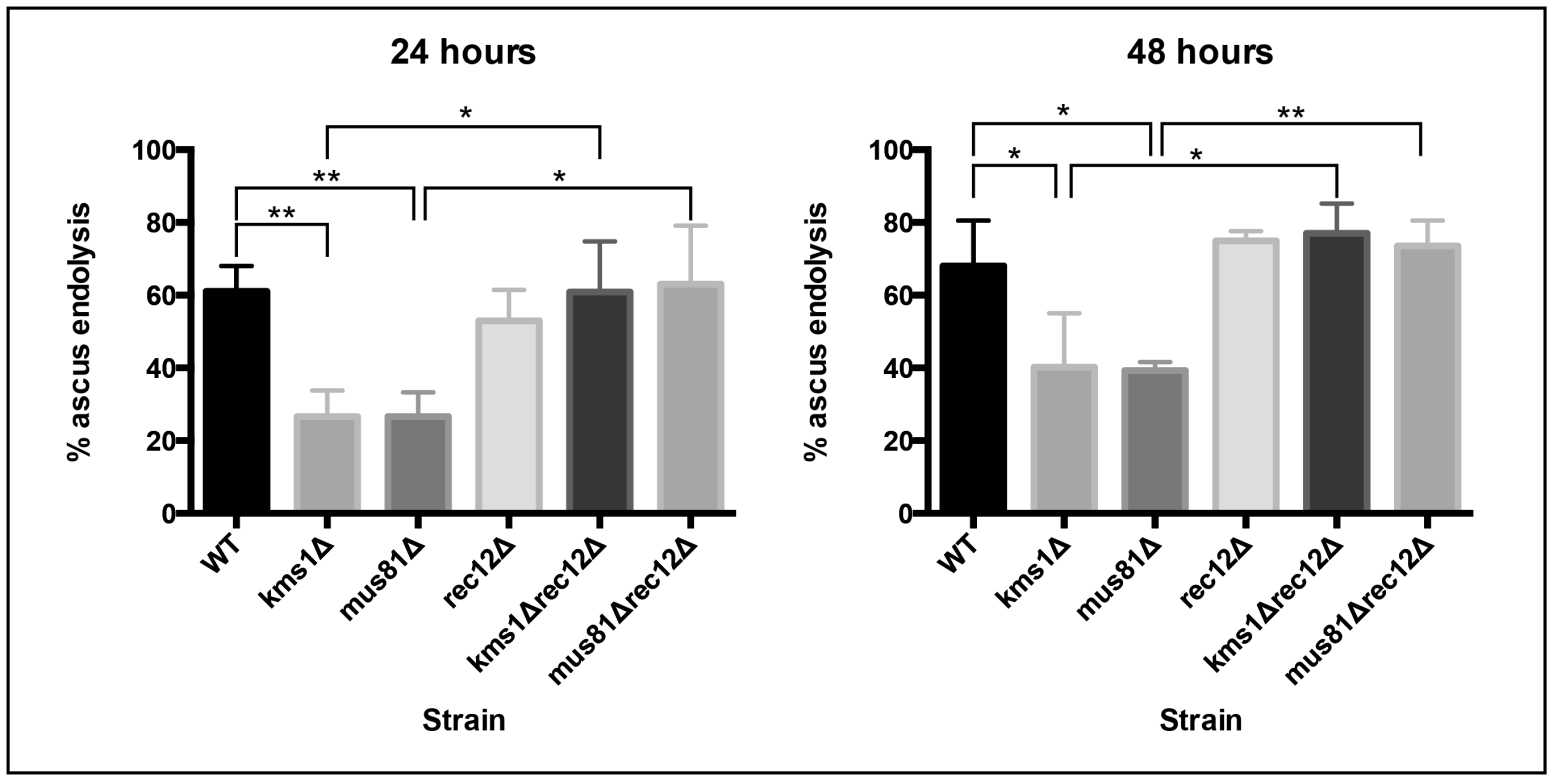
Inhibition of ascus wall endolysis requires meiotic DSB formation. Tetrads arising from meioses defective in the induction of programmed DSBs (*rec12Δ*) break down at rates similar to WT. In combination with the *kms1Δ* and *mus81Δ* alleles, preventing DSB induction can rescue the rate of ascus wall endolysis to WT levels. Analysis was carried out as in Figure 1B. * p<0.025 ** p<0.004

Of the genetic backgrounds we tested, only *kms1Δ* and *mus81Δ* caused lower rates of ascus wall endolysis. In addition to disrupting the characteristic horsetail motion in meiotic prophase, loss of Kms1 also leads to an increase in ectopic recombination and a decrease in allelic recombination [17] while loss of Mus81 inhibits the resolution of meiotic crossovers [26], [27]. These results suggest that persistent recombination intermediates and/or unrepaired DNA double strand breaks (DSBs) may elicit the cascade of events that culminates with slower or absent ascus wall endolysis. Meiotic recombination is initiated by programmed DSBs made by the Spo11 nuclease (Rec12 in *S. pombe*). Without Rec12, programmed DSBs do not occur, thus bypassing any defect in the process of recombination. Despite poor spore viability [28], tetrads arising from a *rec12Δ* [29] mating undergo ascus wall endolysis at rates similar to WT (Figure 2). To test whether DSB formation is required for the *kms1Δ* and *mus81Δ* tetrads to elicit the ascus wall endolysis checkpoint, we analyzed the percentage of tetrads undergoing endolysis in the *kms1Δrec12Δ* and *mus81Δrec12Δ* backgrounds. Preventing the induction of DSBs completely abrogated activation of the checkpoint, leading to normal levels of ascus wall endolysis in the double mutant strains (Figure 2). Thus, it is likely that unrepaired DSBs lead to both a delay in the meiotic divisions and a signal that prevents release of spores from the ascus. In this way, spores arising from an ineffective meiosis are less likely (or slower) to germinate.

### Sensitivity of ascus wall endolysis to meiotic failure depends on the ATR/ATM kinases

Our findings suggested that defects in repair of programmed DSBs might activate a checkpoint that inhibits ascus wall endolysis. During meiosis, such defects lead to the activation of the ATR and ATM kinases to stall meiosis I (the recombination or pachytene checkpoint) and allow for more time to resolve recombination intermediates [5]. To test whether ATR and ATM might play a role in communicating delays or defects in meiotic recombination to the efficiency of ascus wall endolysis, we investigated loss of Rad3 (ATR) and Tel1 (ATM).

**Figure 3:**
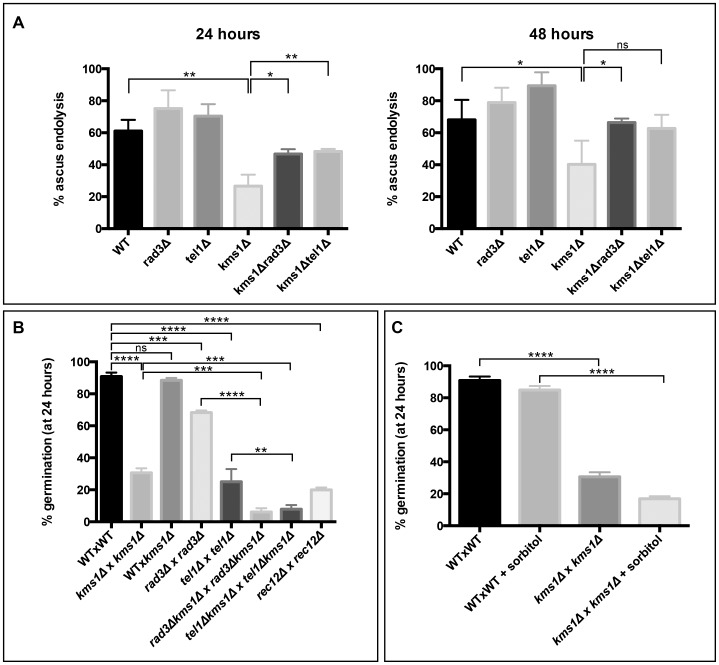
Communication between meiotic success and ascus wall endolysis requires both the ATM and ATR kinases. **A**. The combination of the *rad3Δ* or *tel1Δ* and *kms1Δ* alleles rescues the percentage of ascus wall endolysis seen in the *kms1Δ* alone. Analysis was carried out as in Figure 1B. **B**. Loss of the checkpoint kinases compromises spore health and uncouples ascus wall endolysis from spore quality. Spores were examined by microscopy 24 hours after plating and scored for germination by morphology. Values are the averages of at least three experiments plotted with their standard deviations and p-values were determined by unpaired t-test. The poor germination efficiency of spores from a *kms1Δ* cross arises in meiosis rather than from the *kms1Δ* genotype, as germination of spores from a WT x *kms1Δ* cross was equivalent to WT crosses. Despite increased rates of ascus wall endolysis, both *rad3Δ* and *tel1Δ* crosses showed compromised germination; fewer spores from the *kms1Δrad3Δ* and *kms1Δtel1Δ* combinations germinate than spores from a *kms1Δ* cross. Disruption of meiotic recombination also decreased the percentage of spores that were competent to germinate (*rec12Δ*). The number of tetrads giving rise to colonies was counted four days after visual inspection of ascus wall endolysis. **C**. Poor germination of spores arising from a *kms1Δ* meiosis cannot be rescued by osmotic stabilization on plates containing sorbitol. Analysis was carried out as in **B**. except that spores were plated to YE5S plates containing 1 M sorbitol. * p<0.05 ** p<0.01 *** p<0.0005 **** p<0.0001

Loss of either Rad3 or Tel1 led to a very mild increase in the percentage of tetrads undergoing ascus wall endolysis, although this was only statistically significant for *tel1Δ* crosses at 48 hours (Figure 3A). Nonetheless, this observation suggests several interesting possibilities. First, an increase in ascus wall endolysis relative to WT could indicate that a proportion of WT meioses may be compromised and activate an ATR- and ATM-dependent checkpoint, which prevents ascus wall endolysis. This is consistent with the observations in many other models, including worms and mammals, that meiotic failure occurs at an appreciable rate (see more below) [6]. Further, this result suggests that inhibiting germination of spores arising from a compromised meiosis may promote higher quality progeny, much as the apoptotic pathway removes gametes from the germ line population in higher organisms when meiosis fails. Consistent with this idea, the combination of *kms1Δ* and either *rad3Δ* or *tel1Δ* rescued the percentage of tetrads undergoing ascus wall endolysis to nearly WT levels by 48 hours (Figure 3A). Thus, the activation of the checkpoint preventing endolysis when meiosis fails requires both the ATR and ATM homologues, consistent with a signaling mechanism that may affect both the timing of the meiotic divisions as well as downstream processes such as ascus wall endolysis. By contrast, simultaneous loss of the meiosis-specific kinase Mek1 was not able to suppress the *kms1Δ* phenotype (unpublished data).

### Checkpoint mutants uncouple spore viability and ascus wall endolysis

Since deletion of either Rad3 or Tel1 increased the rates of ascus wall endolysis in the context of the *kms1Δ* background, we were curious about the relationship to spore quality. To examine this, we carried out random spore analysis (RSA), in which the ascus wall is degraded by exogenous enzymes, such as glusulases, to liberate all spores. We then monitored germination of spores 24 hours after plating. Consistent with defects in the progeny of spores arising from a *kms1Δ* meiosis, the majority of spores failed to germinate within 24 hours while 90% of spores arising from a WT meiosis germinated (Figure 3B). The germination defect observed in the progeny derived from a *kms1Δ* cross arises in meiosis rather than reflecting a requirement for Kms1 during germination, as crosses between WT and *kms1Δ* parental strains, in which half of the progeny have the *kms1Δ* genotype, displayed WT levels of germinating spores (Figure 3B). Further, the poor germination of spores arising from a *kms1Δ* cross did not appear to be due to osmotic effects, as growth on plates containing 1 M sorbitol could not suppress the germination defect (Figure 3C).

We next investigated the consequences of inactivating Rad3 and Tel1. The overall quality of spores obtained by RSA arising from crosses lacking these factors were lower than WT, with about 70% of *rad3Δ* progeny germinating within 24 hours with an even more dramatic defect (rates of just over 20%) for *tel1Δ* progeny. We next examined how loss of the checkpoint kinases impacted progeny arising from a *kms1Δ* meiosis. The combination of either *rad3Δ* or *tel1Δ*with *kms1Δ* leads to lower rates of germination (less than 10%) than any of the single alleles. This is in stark contrast to ascus wall endolysis, which is suppressed by deletion of either Rad3 or Tel1 (Figure 3). These results underscore the importance of these checkpoint kinases in ensuring high quality progeny by monitoring several stages of meiosis. Further, in these checkpoint mutant strains ascus wall endolysis becomes uncoupled from progeny quality.

To further test the connection between spore quality and the rate of ascus wall endolysis, we examined progeny arising from a *rec12Δ* cross. Despite poor rates of germination (Figure 3B), tetrads arising from a *rec12Δ* cross undergo ascus wall endolysis at rates similar to WT (Figure 2). Consistent with our analysis of the checkpoint mutants, this suggests that it is not spore quality *per se* that influences ascus wall endolysis, but rather that information from meiosis, most likely the presence of unresolved DNA damage, which suppresses the endolytic machinery.

### Spores from spontaneous endolysis are of higher quality than from chemical endolysis

**Figure 4:**
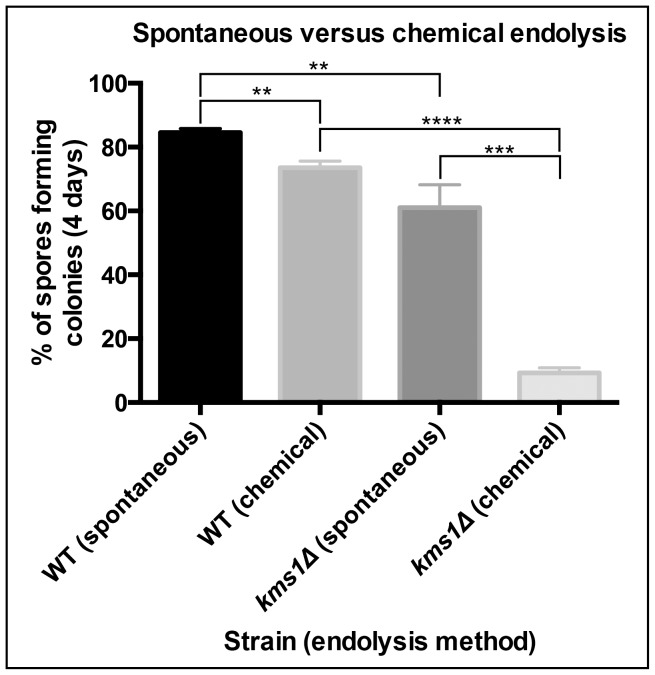
The sensitivity of ascus wall endolysis to meiotic defects increases the fitness of progeny. Viability of spores arising from a *kms1Δ* cross that were released by spontaneous ascus wall endolysis is substantially higher than spore viability calculated from the entire spore population. WT and *kms1Δ* tetrads were allowed to undergo ascus wall endolysis spontaneously or the ascus walls were chemically removed using glusulase, as indicated. Following micromanipulation, the number of colonies arising from individual spores was quantitated after four days. Averages with their standard deviations from at least three replicates of a minimum of 48 spores are shown. p-values were determined by unpaired t-test. ** p<0.005 *** p = 0.0003 **** p<0.0001

Our results suggest that defects arising in a *kms1Δ* meiosis that compromise spore quality prevent ascus wall endolysis in a manner dependent on the ATR/ATM checkpoint. Our data suggest that “chemical” tetrad breakdown, commonly used to examine the genetic requirements for meiotic recombination because it yields large quantities of progeny, might reveal a different quality of progeny than those tetrads that breakdown spontaneously through the ascus wall endolysis pathway that is subject to inputs from meiosis. To test this, we compared the viability of spores from WT and *kms1Δ* crosses that arose from spontaneous and chemical ascus wall endolysis. As shown in Figure 4A, we observe a modest increase in the viability of WT spores liberated spontaneously from the ascus compared to the total spore population released by chemical endolysis. Consistent with our RSA analysis of germination and colony forming ability, only 10% of spores from a *kms1Δ* cross released chemically gave rise to colonies after four days. In stark contrast, nearly 60% of *kms1Δ* spores released naturally from their ascus walls were viable. This suggests that the ascus wall endolysis checkpoint contributes to the preferential release of high quality progeny.

## Discussion

**Figure 5:**
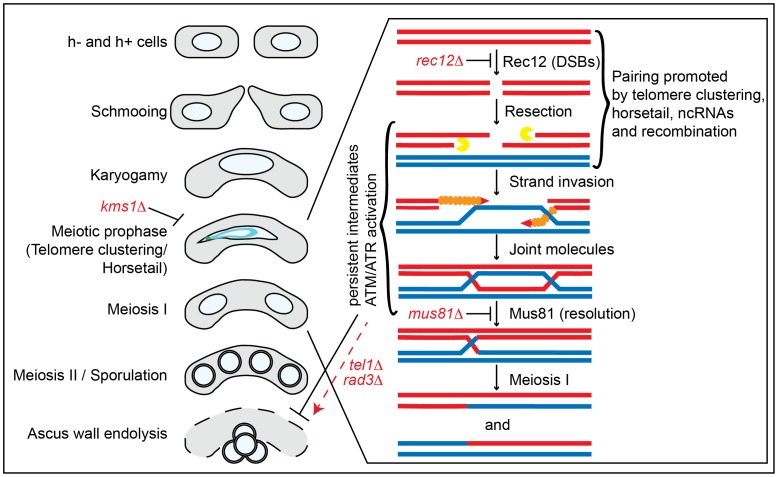
Model for cross-talk between meiosis and ascus wall endolysis. Schematic of meiosis and sporulation. The progression of meiosis in *S. pombe* is outlined on the left. Mating pheromones trigger schmooing behavior, leading to conjugation and nuclear fusion (karyogamy). During meiotic prophase, the telomeric bouquet forms and nuclear oscillations occur (termed horsetail movement) that facilitate homologous chromosome pairing; these events are compromised in *kms1Δ*meioses. Chromosome pairing is also promoted by ncRNAs [37] and recombined chromatids (see below). Meiotic recombination, outlined on the right, is triggered by Rec12-dependent DNA double strand breaks (DSBs). The recombination machinery drives formation of joint molecules between sister chromosomes, while Mus81 and associated factors promote resolution of Holliday junctions. We hypothesize that unresolved DSB repair intermediates persist when chromosome pairing (*kms1Δ*) or Holliday junction resolution (*mus81Δ*) is compromised. These intermediates are sensed by the ATR and ATM kinases, which then repress ascus wall endolysis. Without the checkpoint kinases Tel1 or Rad3, spores are released despite persistent recombination intermediates. Meiosis I leads to separation of the recombined sister chromosomes followed by segregation of sister chromatids in Meiosis II to give rise to haploid progeny competent to undergo spore formation. After nutrients are available, the ascus wall undergoes endolysis and spores germinate.

Here we suggest the existence of a pathway capable of coupling ascus wall endolysis to meiotic success in *S. pombe*. Our data are consistent with a model in which unrepaired DNA damage, sensed by the DNA damage kinases ATR (Rad3) and ATM (Tel1), restrains the activation of glucanase(s) to delay or inhibit ascus wall endolysis (Figure 5). In such a model, glucanase activity would have to be cell-autonomous. Interestingly, the *A. fumigatus* Eng2 orthologue has been found to be a GPI-anchored protein [30], suggesting that it might only act on the ascus wall of the tetrad in which it is generated. It does not appear that Eng2 expression is limited to meiosis (unpublished data and [14]), suggesting that secretion or activation of Eng2 (and/or Agn2) might be a regulated event. Further, Eng2 and/or Agn2 could potentially serve to integrate multiple inputs, of which meiotic success may be just one, in order to influence ascus wall endolysis. Interestingly, proposed coupling of meiotic surveillance to downstream developmental events displays striking parallels to a pathway in *Drosophila* in which unrepaired DSBs activate the ATR/ATM homologue mei-41, which in turn suppresses translation of the Gurken mRNA, leading to defects in dorsoventral patterning during oogenesis [31], [32].

We have shown that activation of the ascus wall endolysis checkpoint acts in concert with other meiotic checkpoints in *S. pombe*. Therefore, it is possible that the relatively weak and short-lived recombination checkpoint response in *S. cerevisiae* [33] and *S. pombe* [34] might be mitigated to some extent by a bias in the rates of release of spores according to their meiotic history. Why would there be a selective advantage for a unicellular eukaryote to prevent release of low-quality progeny? Most non-laboratory yeast strains in their native environment display cooperative behaviors, growing in biofilms or flocs [35] where induction of meiosis in response to growth conditions could occur simultaneously in a large number of essentially clonal cells. Thus, retention of spores arising from a compromised meiosis in the ascus might bias resources towards high quality progeny or remove less fit individuals from future mating events. Interestingly, it has been reported that the basidiomycete *Coprinus cinereus* induces apoptosis of spores in mutants that fail in synapsis during meiotic prophase, suggesting that diverse fungi may adopt different strategies to ensure high quality progeny [36].

## Supporting Information

Table S1 ***S. pombe*** **strains used in this study.**
(DOCX)
