# Supplementary material for: A Quality Control Mechanism Linking Meiotic Success to Release of Ascospores
Source: PLoS One. 2013 Dec 2;8(12):e82758. doi: 10.1371/journal.pone.0082758 (PMC3846778; doi:10.1371/journal.pone.0082758)
Supplement: Table S1 — S. pombe strains used in this study. (DOCX) [file pone.0082758.s001.docx]

| Strain | Genotype | Source |
| --- | --- | --- |
| MKSP112 | h- ura4-D18 leu1-32 taz1::ura4+ | YGRC; FY14161 |
| MKSP125 | h+ leu1-32 his2-245 bqt1::LEU2 | CRLg11 (Chikashige et al, 2006; [25]) |
| MKSP126 | h- ura4-D18 leu1-32 lys1-131 ade6-M216 bqt2::ura4+ | CRLi55 (Chikashige et al, 2006; [25]) |
| MKSP200 | h+ ura4-D18 leu1-32 ade6-M21? | This work, derived from PN#1842 (Nurse lab) |
| MKSP201 | h- ura4-D18 leu1-32 ade6-M21? | This work, derived from PN#1842 (Nurse lab) |
| MKSP208 | h- ura4-D18 leu1-32 ade6-M21? kms1::KanR | This work |
| MKSP209 | h+ ura4-D18 leu1-32 ade6-M21? kms1::KanR | This work |
| MKSP370 | h+ ura4-D18 leu1-32 mus81::LEU2 | YGRC, FY18429 |
| MKSP415 | h- ura4-D18 leu1-32 mus81::NatR | This work |
| MKSP543 | h90 ura4-D18 leu1-32 rec12-152 meu13::GFP | YGRC; FY16696 (rec12-152 is a deletion allele - Lin and Smith, 1994; [29]) |
| MKSP633 | h+ ura4-D18 leu1-32 kms1::KanR rad3::ura4+ | This work |
| MKSP634 | h- ura4-D18 leu1-32 kms1::KanR rad3::ura4+ | This work |
| MKSP635 | h+ ura4-D18 leu1-32 kms1::KanR tel1::LEU2 | This work |
| MKSP636 | h- ura4-D18 leu1-32 kms1::KanR tel1::LEU2 | This work |
| MKSP674 | h+ ura4-D18 leu1-32 rad3::ura4+ | This work, derived from FY14087 (YGRC) |
| MKSP675 | h- ura4-D18 leu1-32 rad3::ura4+ | This work, derived from FY14087 (YGRC) |
| MKSP676 | h+ ura4-D18 leu1-32 tel1::LEU2 | This work, derived from FY7799 (YGRC) |
| MKSP677 | h- ura4-D18 leu1-32 tel1::LEU2 | This work, derived from FY7799 (YGRC) |
| MKSP861 | h+ ura4-D18 leu1-32 taz1::ura4+ | Derived from MKSP112 |
| MKSP1034 | h- ura4-D18 leu1? ade6? rec12::ura4+ kms1::KanR | This work |
| MKSP1035 | h+ ura4-D18 leu1? ade6? rec12::ura4+ kms1::KanR | This work |
| MKSP1038 | h? ura4-D18 mus81::KanR rec12::ura4+ | This work |
| MKSP1039 | h? ura4-D18 mus81::KanR rec12::ura4+ | This work |
| MKSP1219 | h- ura4? leu1-32 his2? bqt1::LEU2 | This work, derived from MKSP125 |
| MKSP1220 | h+ ura4-D18 leu1-32 lys1? ade6-M21? bqt2::ura4+ | This work, derived from MKSP126 |
